# Epidemiological Study and Genetic Diversity Assessment of Porcine Epidemic Diarrhea Virus (PEDV) in Yunnan Province, China

**DOI:** 10.3390/v17020264

**Published:** 2025-02-14

**Authors:** Pei Zhu, Hong Yuan, Xianghua Shu, Xue Li, Yaoxing Cui, Lin Gao, Rui Yan, Taoying Yu, Chunlian Song, Jun Yao

**Affiliations:** 1 Yunnan Tropical and Subtropical Animal Virus Diseases Laboratory, Yunnan Animal Science and Veterinary Institute, Kunming 650224, China; zpcau@sina.com (P.Z.); 13987123280@163.com (L.G.); 2 College of Animal Medicine, Yunnan Agricultural University, Kunming 650201, China; yh199600000@163.com (H.Y.); ynndsxh@ynau.edu.cn (X.S.); x2845132072@163.com (X.L.); yaoxin773@163.com (Y.C.); 2011009@ynau.edu.cn (C.S.); 3 Menglian County Animal Disease Prevention and Control Center, Menglian 665899, China; mlyanrui@163.com; 4 Gongshan County Animal Disease Prevention and Control Center, Gongshan 673599, China; ytyytl123@163.com

**Keywords:** PEDV, diarrhea-causing viruses, epidemiology, genetic diversity, co-infection

## Abstract

Porcine epidemic diarrhea virus (PEDV) is a highly contagious pathogen responsible for devastating enteric disease and lethal watery diarrhea, leading to significant economic losses in the global swine industry. Understanding the epidemiology and genetic diversity of PEDV over the past decade is crucial for the effective prevention and treatment of porcine epidemic diarrhea. In this study, 1851 fecal samples were collected from pigs exhibiting diarrhea symptoms across 11 cities in Yunnan Province between 2013 and 2022. The prevalence of PEDV, along with other common swine diarrhea viruses, including porcine transmissible gastroenteritis virus (TGEV), porcine rotavirus (PoRV), porcine Sapporo virus (PoSaV), porcine stellate virus (PaStV), and porcine delta coronavirus (PDCoV) was assessed using a polymerase chain reaction (PCR) assay. The results revealed a total detection rate of 52.94% (980/1851) for the six viruses, with PEDV accounting for 25.93% (480/1851) of cases. Further analysis showed that weaned piglets were more susceptible to PEDV than fattening pigs, with the highest prevalence observed in spring (61.52%, 275/447) and the lowest in summer (12.68%, 97/765). Dual infections were also identified, with PEDV + PoSaV being the most common combination (2.81%, 52/1851), followed by PEDV + PoRV, with a detection rate of 1.67% (31/1851). Phylogenetic analysis of the PEDV S genes revealed that the 28 epidemic strains in Yunnan Province shared a nucleotide sequence homology from 91.4% to 98.4% and an amino acid sequence homology ranging from 85.6% to 99.3%. All strains were classified as GII variant strains. This study provides a comprehensive overview of the epidemiology of PEDV and its co-infection patterns with other common diarrhea-causing viruses in the swine herds of Yunnan Province over the past decade. These findings offer valuable insights for the development of effective prevention and control strategies to mitigate the impact of PEDV and other enteroviruses on the swine industry in Yunnan Province.

## 1. Introduction

Porcine epidemic diarrhea (PED) is a highly contagious and acute intestinal disease caused by the porcine epidemic diarrhea virus (PEDV). Characterized by enteritis and severe watery diarrhea, PED affects pigs of all ages, with mortality rates reaching up to 100% in suckling piglets [1,2]. This disease poses significant threats to swine populations, leading to widespread epidemics and substantial economic losses [1]. PEDV belongs to the genus Alphacoronavirus within the family Coronaviridae and the order Nidovirales. It is classified into two major genotypes: GI (classical) and GII (variant). These genotypes have further diverged into five subgroups: GIa, GIb, GIIa, GIIb, and GIIc, with the GIIc subgroup also referred to as the S-INDEL strain [3,4]. Classical PEDV strains, such as CV777 and DR13, are representative of the GI genotype [4].

The PEDV genome is approximately 28 kb in length and comprises seven open reading frames: ORF1a, ORF1b, and ORF2-6. Four structural proteins are encoded by ORF2 and ORF4-6: the spike (S) protein, the membrane (M) protein, the envelope (E) protein, and the nucleocapsid (N) protein. Additionally, the ORF3 gene encodes a single accessory protein known as ORF3 [3,4]. The S protein, consisting of 1383 amino acids, is a key surface immunogenic protein of PEDV. It plays a critical role in cell fusion, viral virulence, and the induction of neutralizing antibodies [5]. Due to its high variability, the S protein is widely used for the phylogenetic analysis of PEDV isolates, making it an important target for understanding the evolution and diversity of the virus [6].

PEDV was first reported in England in 1977 [7] and subsequently isolated in China in 1984 [8]. Since 2010, GII genotype strains have become predominant worldwide [1]. In China, the emergence of highly virulent PEDV strains in 2010 resulted in piglet mortality rates of 100%, causing devastating economic losses [9]. Similarly, a PED outbreak in the United States and neighboring countries (Canada and Mexico) in April 2013 led to the death of over eight million piglets in the US alone [10,11]. Shortly thereafter, highly virulent PEDV strains were reported in Japan, South Korea, Vietnam, and Thailand [1]. Taiwan and China’s mainland experienced a PED outbreak in late 2013 [12]. Meanwhile, the PEDV S-INDEL strain (GIIc) appeared in Germany in 2014 and has since become endemic in many European countries [1]. Yunnan Province is one of the major pig-raising regions in China. However, despite its importance in swine production, there have been relatively few studies on the epidemiological situation of PEDV in this area [13,14]. Additionally, the existing studies are limited by small sample sizes and short observation periods, which restrict the comprehensiveness of the findings.

In addition to PEDV, swine enteric coronaviruses (SECoVs) include porcine transmissible gastroenteritis virus (TGEV) and porcine delta coronavirus (PDCoV). Other porcine diarrhea viruses, such as porcine rotavirus (PoRV), porcine sapovirus (PoSaV), and porcine astrovirus (PAstV), have also been reported as prevalent in Yunnan Province [3,15]. The similarity of clinical symptoms and pathological findings among these viral infections complicates clinical diagnosis [16]. Furthermore, co-infections and secondary infections are also frequently observed. For instance, a survey of swine diarrhea samples in Jiangxi Province from 2012 to 2015 revealed an infection rate of 33.71% for PDCoV, with a PDCoV/PEDV co-infection rate of 19.66% [17]. In the United States, the PDCoV/PoRV co-infection rate in swine herds was reported to be 30% [18]. Triple infections involving PEDV, PoRV, and PDCoV are also common [19]. Similarly, between 2021 and 2022, 77 out of 306 diarrhea samples collected from 54 farms in India showed mixed infections with PAstV and PoRV [20]. However, few studies have yet reported on the co-infection status of these porcine diarrhea viruses in the Yunnan region.

Hence, to investigate the epidemiology of porcine diarrhea viruses in Yunnan Province, we collected fecal samples from pigs exhibiting diarrhea symptoms across the region, and the presence of six porcine diarrhea viruses (PEDV, TGEV, PoRV, PoSaV, PaStV, and PDCoV) was detected using a polymerase chain reaction (PCR) assay. Additionally, a phylogenetic analysis of PEDV S genes was performed to further explore the epidemiology and genetic diversity of PEDV genotypes. This study provides valuable insights that can inform the development of effective prevention and control strategies to mitigate the impact of PEDV and other enteric viruses on the swine industry in Yunnan Province.

## 2. Materials and Methods

### 2.1. Sample Collection

Fecal samples were collected from pigs exhibiting diarrhea symptoms using a sterile swab. The samples were immediately placed in sterile containers and transported to the Yunnan Tropical and Subtropical Animal Virus Diseases Laboratory in an ice box. Upon arrival at the laboratory, the samples were mixed with phosphate-buffered saline (Gibco, Beijing, China) at a 1:10 ratio. The mixtures were then centrifuged at 10,000× *g* for 15 min at 4 °C to separate the supernatants, which were subsequently stored at −80 °C for further laboratory analysis.

### 2.2. RNA Extraction and RT-PCR Analysis

The viral RNA genome was extracted from the supernatants using the MiniBEST Viral RNA/DNA Extraction Kit Ver. 5.0 (Takara Bio, Dalian, China), following the manufacturer’s protocol. Specific primers for TGEV-S, PoRV-VP6, PoSaV-VP1, PaStV-0RF2, and PDCoV-N were designed using NCBI Blast according to reference sequences (GenBank Nos. AJ271965.2, FJ617209, KX688107, JX556690, and MN942260, respectively) (Table 1). The extracted RNA was reverse-transcribed into complementary DNA (cDNA) using the PrimeScriptTM One-Step RT-PCR Kit (Takara Bio, Dalian, China), whose system included 2 μL of the mix, 25 μL of a step buffer, 1 μL of the forward primer at a 0.4 μM concentration, 1 μL of the reverse primer at a 0.4 μM concentration, 1 μL of the template, and 19 μL of dH2O. The RT-PCR procedure was conducted under the following conditions: 50 °C for 30 min and 94 °C for 2 min, followed by 35 cycles of 94 °C for 30 s, 56 °C for 30 s, and 72 °C for 60 s, with a final extension step at 72 °C for 7 min. After amplification, the reaction mixtures were analyzed using 1% agarose gel electrophoresis, and the expected DNA products were sequenced by Shanghai Shenggong Bioengineering Co., Ltd. (Shanghai, China).

### 2.3. PCR Amplification and Sequencing of S Genes of PEDV

The RNA of PEDV was extracted from supernatants using a Viral RNA Mini Kit (Takara Inc.), and cDNA was obtained using a TaKaRa One-Step RT-PCR Kit with random primers. Five primers (Table 1) with amplification regions covering the entire PEDV S gene were designed based on the PEDV whole-gene sequence (GenBank No. AF353511) to obtain the S gene sequence of the PEDV strains. The PCR procedure was carried out under the following conditions: pre-denaturation at 94 °C for 3 min, followed by 35 cycles of denaturation at 94 °C for 30 s, annealing at 60 °C for 30 s, and extension at 72 °C for 120 s, with a final extension step at 72 °C for 10 min. The reaction mixtures were analyzed using agarose gel electrophoresis following amplification. Positive products were purified using a MiniBEST Agarose Gel DNA Extraction Kit (TaKaRa Bio, Dalian, China) according to the manufacturer’s instructions. The purified DNA fragments were then ligated into the T-vector pMD18 (TaKaRa Bio, Dalian, China) and used to transform competent *Escherichia coli DH5a* cells. Transformed colonies were screened using the respective fragment amplification primers. For each fragment, two positive clones were selected and sent for sequencing at Shanghai Shenggong Bioengineering Co., Ltd.

### 2.4. Sequence Characterization and Phylogenetic Analysis of PEDV S Genes

The PEDV S gene sequences of representative Yunnan epidemic strains were obtained and assembled using DNAMAN (v6.0). The amino acid sequences were predicted using the ORF analysis software from the NCBI (https://www.ncbi.nlm.nih.gov/ accessed on 1 February 2025). The sequence characteristics were analyzed based on the size of the complete gene, untranslated regions, and open reading frames and their coding amino acids. Comparative analyses of the full-length sequences of the PEDV S gene and their encoded amino acids were conducted to investigate the phylogenetic relationships between the representative Yunnan PEDV strains and other PEDV strains. The full-length sequences of the PEDV S gene and the corresponding amino acid sequences of reference PEDV strains were downloaded from GenBank. Detailed information about the downloaded full-length sequences is listed in Table 2.

The nucleotide and predicted amino acid sequences were aligned using MAFFT software(version 7). Nucleotide and amino sequence similarities were calculated using BioEdit (v 7.1.3.0). A phylogenetic tree based on the nucleotide sequences of the PEDV S gene was constructed using the neighbor-joining (NJ) method with 1000 bootstrap replicates in MEGA 7.0.

Recombination analysis was conducted using 7 methods provided by the recombination detection software RDP (version 4.1.3), including RDP, GENECONV, BootScan, Max-Chi, Chimaera, SiScan, and 3Seq. The threshold *p*-value for detecting recombination was set to 0.01. The S gene sequences of the representative strains were compared with sequences downloaded from GenBank (Table 2). When more than 5 methods detected recombination signals, further analysis was performed using Simplot software (version 3.5.1).

## 3. Results

### 3.1. Detection and Analysis of Diarrhea Viruses in Fecal Samples from Yunnan Province

**Figure 1:**
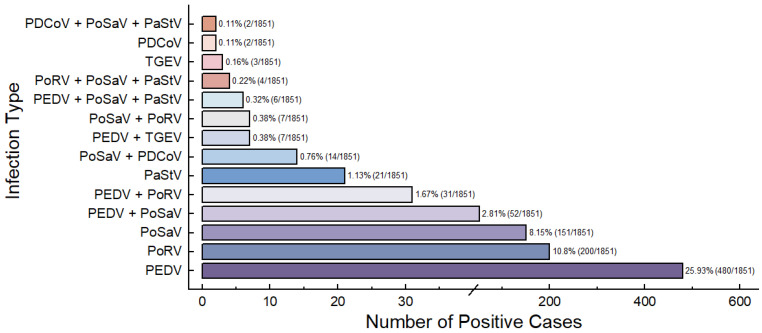
Detection rates of different diarrhea virus infection types in 1851 samples from Yunnan Province.

A total of 1851 fecal samples were collected over the past decade from 11 cities in Yunnan Province, including Yuxi, Dali, Kunming, Qujing, Honghe, Wenshan, Lijiang, Lincang, Chuxiong, Baoshan, and Zhaotong. Detailed information about the collected samples is provided in Table 3. The overall detection rate for the six diarrhea virus in these samples was 52.95% (980/1851). The detection rates for individual viruses were as follows: PEDV: 25.93%, 480/1851; TGEV: 0.16%, 3/1851; PoRV: 10.8%, 200/1851; PoSaV: 8.15%, 151/1851; PaStV: 1.13%, 21/1851; and PDCoV: 0.11%, 2/1851. Among the positive samples, 46.30% (857/1851) were identified as mono-infections, while 6.65% (123/1851) were classified as mixed infections. The majority of mixed infections involved dual or triple infections, with no quadruple infections detected. The most prevalent dual infection was PEDV + PoSaV, with a detection rate of 2.81% (52/1851), followed by PEDV + PoRV at 1.67% (31/1851). Triple infections were dominated by PEDV + PoSaV + PaStV (0.32%, 6/1851), PDCoV + PoSaV + PaStV (0.11%, 2/1851), and PoRV + PoSaV + PaStV (0.22%, 4/1851) (Figure 1).

### 3.2. Distribution of PEDV in Yunnan Province

**Figure 2:**
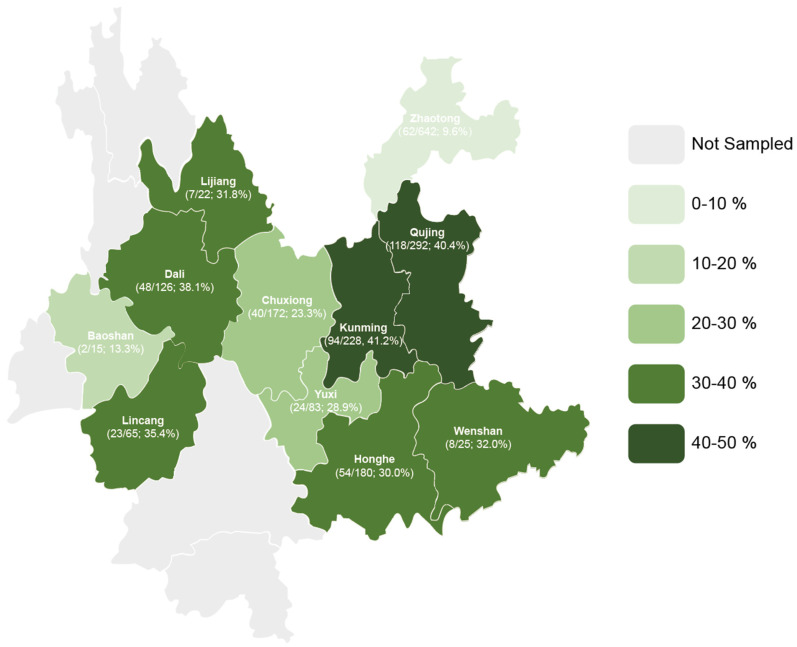
Geographical distribution of detected PEDV in Yunnan Province.

The PCR results revealed that PEDV was prevalent in pig herds across 11 regions in Yunnan Province. The highest detection rates were observed in Kunming (41.2%, 94/228) and Qujing (40.4%, 118/292), while Zhaotong had the lowest detection rate at 9.6% (62/643). Among the other regions, the prevalence rates ranged from 13.3% (2/15) in Baoshan to 38.1% (48/126) in Dali (Figure 2).

The detection rate of PEDV infection in fattening pigs was 8.64% (93/1076), which was significantly lower than that in weaned piglets (62.3%, 321/515) and suckling sows (52.3%, 136/260). Seasonal analysis indicated that PEDV infections were most prevalent in spring, with a detection rate of 61.5% (275/447). This was followed by winter with 33.1% (129/390), fall with 23.7% (59/249), and summer with 12.7% (97/765). Detailed information about the population and seasonal distributions of PEDV is provided in Table 4.

### 3.3. Sequencing and Phylogenetic Analysis of PEDV S Gene

**Figure 3:**
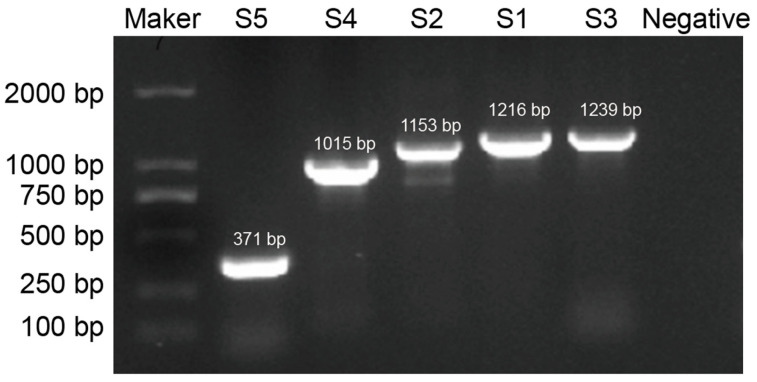
Gel picture of the PEDV S genes from a representative strain.

A representative sample of 28 PEDV-positive strains was selected from different regions and time points. These strains were amplified, cloned, and sequenced, resulting in the complete S gene sequences of all 28 strains Figure 3. Evolutionary analysis revealed that the nucleotide sequence homology of the S gene among the 28 Yunnan epidemic strains ranged from 91.4% to 98.4%, while the amino acid sequence homology ranged from 85.6% to 99.3% when compared with the domestic and international reference strains from GenBank shown in Table 2. 

**Figure 4:**
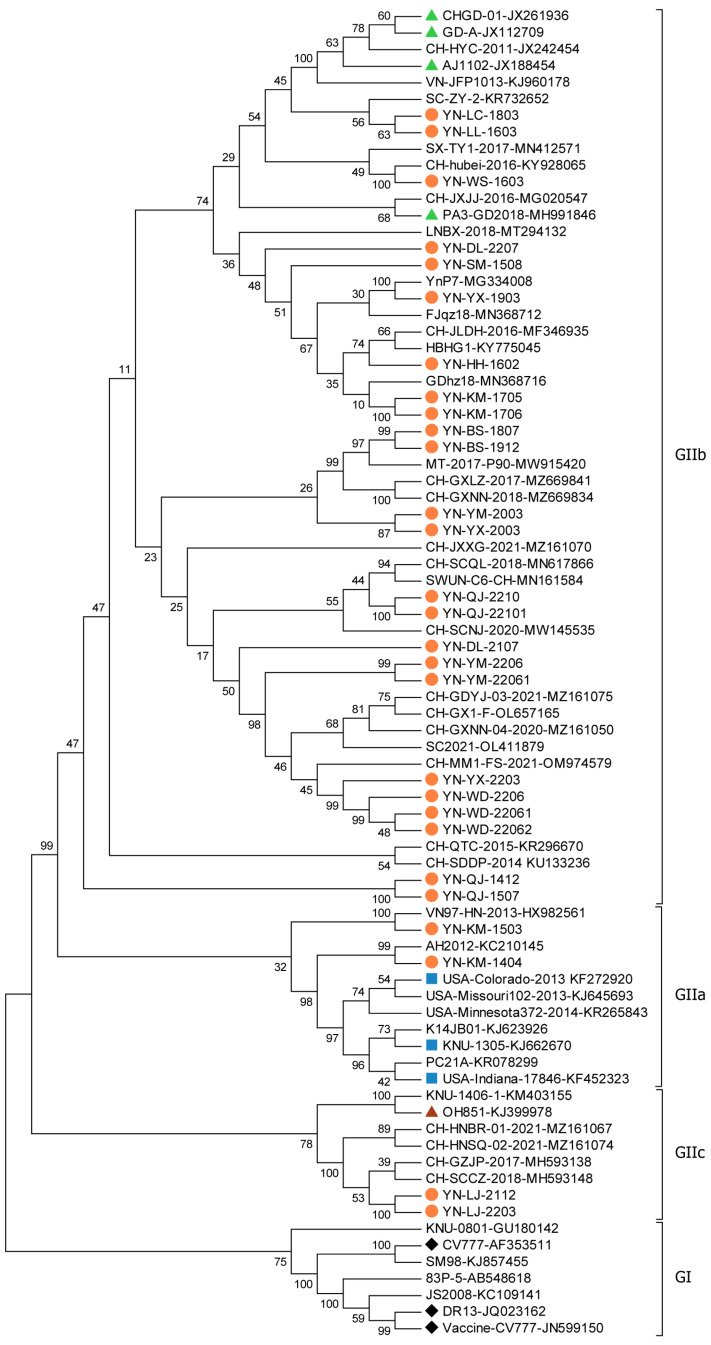
Phylogenetic tree based on the PEDV S genes of the 28 representative PEDV strains, created using the neighbor-joining (NJ) method. 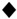: classical strain GI group; 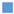: variant GIIa subgroup; 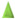: variant GIIb subgroup; 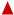: S-INDEL subgroup; 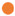: representative PEDV strains.

When compared with the classical strain CV777 from the PEDV subgroup GIa, the S gene and its corresponding amino acid sequences showed homology ranges of 86.2% to 89.8%. In contrast, the homology ranged from 90.1% to 96.2% when compared with the non-S-INDEL strain AJ1102 from subgroup GIIb. Phylogenetic analysis placed 20 of the 28 Yunnan strains within the same evolutionary branch, closely related to Group GII strains (including AJ1102, GD-A, and OH851) and more distantly related to Group GI strains (including CV777 and DR13). Notably, YN-KM-1404 and YN-KM-1503 were genetically similar to the KNU-1305 and USA/Colorado/2013 strains within subgroup GIIa. Additionally, the strains YN-LJ-2112 and YN-LJ-2203 were more closely related to the strain OH851, belonging to subgroup GIIc. The remaining 24 strains showed closer genetic relationships to AJ1102 and CHGD-1, classifying them within subtype GIIb (Figure 4).

Compared with the classical vaccine strain CV777, the S gene of the 28 Yunnan strains exhibited 73 amino acid changes, including 69 mutations, 2 insertions, and 2 deletions. For instance, the amino acid changes included PS→LF at positions 462–463, SL→LF at positions 1313–1314, the deletion of Q at positions 721 and 1285, and the insertion of L and I at positions 945 and 1284 (Table 5). When compared with the variant vaccine strain AJ1102, the 28 Yunnan strains displayed 11 amino acid mutations but no insertion or deletion (Table 6). However, some prevalent Yunnan strains also exhibited amino acid insertions and deletions in several regions when compared with both the classical vaccine strain CV777 and the variant vaccine strain AJ1102.

### 3.4. Recombinant Analysis of PEDV S Gene Sequences

**Figure 5:**
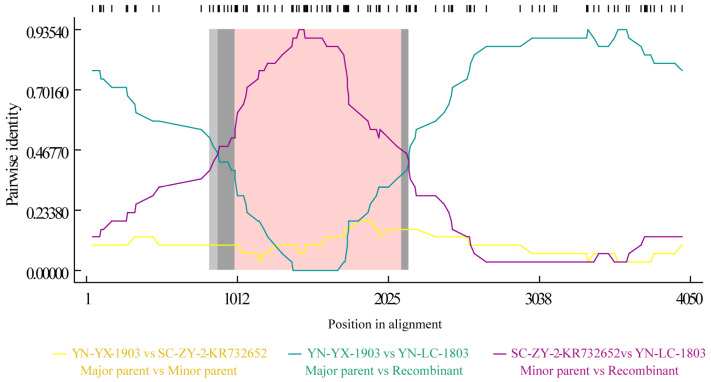
Recombination plot of RDP 4 genes.

**Figure 6:**
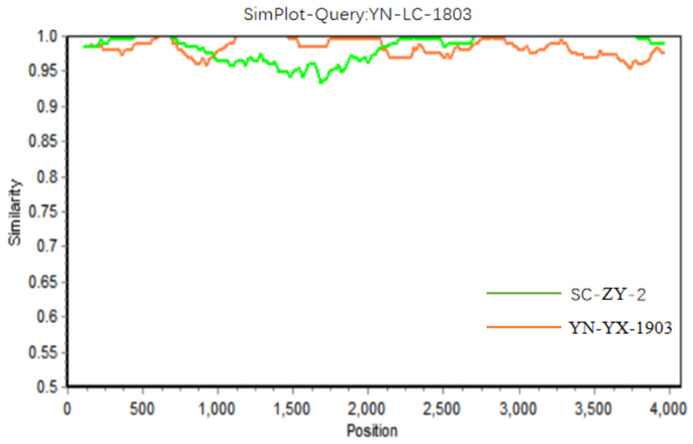
Simplot gene recombination plot.

YN-LC-1803 exhibited a recombination signal detected by the seven different methods. The predicted genetic recombination fragments were located between positions 1007 and 2132, indicating that YN-LC-1803 may be a recombinant strain. The major parental strain was identified as “YN-YX-1903”, while the minor parental strain was “SC-ZY-2-KR732652” (Figure 5). Simplot software confirmed the similarity results and aligned with the findings from RDP4 (Figure 6).

Phylogenetic analysis of the S gene of the 28 Yunnan PEDV strains revealed that all strains belonged to the GII type. Among these, twenty-four strains were classified as the GIIb subtype, two as the GIIa subtype, and two as the S-INDEL subtype. Additionally, a recombination event was detected in YN-LC-1803.

## 4. Discussion

PEDV has been endemic across most continents since its emergence in the 1970s and remains a critical cause of piglet mortality, particularly in suckling piglets, where mortality rates can reach up to 100% [4]. Wang et al. reported a PEDV positivity rate of 83.03% (137/165) in samples collected from 41 PEDV-positive farms across 18 provinces in China [21]. Similarly, Zhang et al. analyzed 149,869 clinical samples from diarrheic pigs in eight provinces of China between 2011 and 2021. Their findings revealed that PEDV was the dominant pathogen, with an infection rate exceeding 40% over 11 years. However, the pathogenesis of porcine diarrhea in China is complex, with frequent co-infections and multiple infections, and mixed infections involving TGEV, PoRV, and PDCoV were also widely observed [1], and the similarity in the clinical symptoms and pathological anatomy among these viral infections complicates clinical diagnosis, necessitating laboratory testing to identify the causative agent.

PEDV is frequently co-infected with other diarrhea-causing viruses in pigs. For example, Qi et al. analyzed 217 PEDV-positive diarrhea samples from 17 US states between 2015 and 2016 using second-generation sequencing and identified nine additional RNA viruses co-infecting with PEDV [22]. In 2020, Liu et al. tested 202 acute diarrhea samples from piglets in Yunnan Province and found that 56 of the 71 PoSaV-positive samples (78.9%) were co-infected with other diarrhea viruses [23]. In our study, 1851 samples from 11 cities in Yunnan Province were tested for six porcine diarrhea viruses, including PEDV, TGEV, PoRV, PoSaV, PaStV, and PDCoV, using RT-PCR. Of these, 980 samples (52.95%) tested positive for at least one virus. PEDV was the most dominant pathogen, with a detection rate of 25.93% (480/1851). The detection rates for PoRV, PoSaV, PaStV, TGEV, and PDCoV were 10.8% (200/1851), 8.15% (151/1851), 1.13% (21/1851), 0.16% (3/1851), and 0.11% (2/1851), respectively. Double and triple infections were observed, with PEDV + PoSaV being the most common mixed infection, detected in 2.81% (52/1851) of samples (Figure 1). These findings confirm that PEDV is the primary pathogen causing diarrhea in pigs in Yunnan Province.

From 2019 to 2021, Yang et al. analyzed 2319 porcine serum samples from 284 large-scale pig farms in eight regions of Yunnan Province and found an average PEDV antibody positivity rate of 31.65% [24], aligning with the results of our study. However, in Liu’s study, PoSaV was the leading cause of acute diarrhea outbreaks in Yunnan piglets in 2020, with an infection rate of 35.2% (71/202) [23]. This discrepancy may be attributed to differences in feeding management and epidemiology factors. Overall, Yunnan Province should strengthen the monitoring and control of PEDV, PoSaV, TGEV, PaStV, PoRV, and PDCoV. These six porcine diarrhea viruses occur year-round, with outbreaks peaking in winter and spring. PEDV is particularly infectious during these seasons, with infection rates of 61.5% (275/447) in spring and 33.1% (129/390) in winter. The virus predominantly infects suckling piglets and lactating sows, with infection rates of 62.3% (321/515) and 52.3% (136/260), respectively (Table 4), consistent with previous reports [25,26,27].

The S gene is the primary virulence gene of PEDV, and mutations in this gene significantly impact PEDV genotyping and virulence [28]. The continuous emergence of new, highly virulent PEDV strains has caused substantial economic losses worldwide. Successive mutations in the S gene have led to significant genetic differences between the current predominant strain (PEDV GII) and the classical GI strain, as well as the vaccine strain CV777. Neither the CV777-inactivated vaccine nor the attenuated live vaccine provides effective protection against these new strains [6,25]. In this study, all 28 representative PEDV strains from Yunnan Province belonged to the type GII genotype, indicating that the GII variant has been dominant in the region in recent years. Amino acid homology analysis revealed low similarity between the GII strains and the GI type represented by the CV777 strain but high similarity to the GII variant vaccine strain AJ1102 (Figure 4). This suggests that classical vaccines may not provide adequate protection against PEDV, underscoring the need for updated vaccines.

One strain, YN-LC-1803, was identified as a recombinant strain, with YN-YX-1903 as the major parent and SC-ZY-2 as the minor parent, both belonging to the PEDV GIIb subtype. This finding suggests that recombination and mutation events occur between PEDV in Yunnan and other regions of China. The development of novel, protective vaccines remains a top priority for controlling PED. Since the emergence of highly virulent PEDV variants in China in 2010 [9], the GIIb subtype has been the dominant strain. A GIIb-targeted vaccine, represented by the AJ1102 strain, has been developed and marketed. However, recent epidemiological data indicate a decline in GIIb subtype strains and a rise in GIIa subtype strains over the past two years [29,30], likely due to the widespread use of GIIb-targeted vaccines. The evolutionary pattern of the GII viruses also shows geographic variation. For example, they evolve steadily in South Korea but undergo frequent recombination in China [1].

Two of the twenty-eight Yunnan epidemic strains in this study belonged to the S-INDEL subtype. Lee et al. analyzed the genome of S-INDEL PEDV and found that it likely arose from recombination events between classical and mutant strains [27,31]. The S protein of S-INDEL PEDV closely resembles that of the classical strain, resulting in lower virulence and pathogenicity compared to non-S-INDEL PEDV strains.

## 5. Conclusions

Collectively, this study demonstrated that PEDV was the primary pathogen causing diarrhea in pigs in Yunnan Province from 2013 to 2022. Other viruses, including PoRV, PoSaV, TGEV, PaStV, and PDCoV, were also prevalent and often co-infected with PEDV. The GII genotype remains dominant according to the phylogenetic analysis of 28 representative Yunnan PEDV strains, with the GIIb subtype prevailing, though the GIIa and S-INDEL subtypes are also present. By analyzing 1851 diarrhea samples collected over a broad time frame and geographic area, this study provides a comprehensive overview of porcine diarrhea epidemiology in Yunnan Province. These findings clarify the role of PEDV and co-infections in swine populations over the past decade and lay a foundation for future epidemiological studies and vaccine development.

## Figures and Tables

**Table 1:** Primers used in the polymerase chain reaction amplification reaction.

Target Genes	Sequence (5′-3′)	Product Size (bp)
*PEDV-S1*	CTTCCAACACTCAGCCTACC	1216
	CAGCATCCAACAAACCGAGA	
*PEDV-S2*	CCTCAGATCCTCATTTAGCC	1153
	TAGAAGAAACCAGGCAACTC	
*PEDV-S3*	GCATTTTGGCAGGTGTTTAT	1239
	GAACATCGGCTGAAAGAATG	
*PEDV-S4*	CAATTGCTTGCTGAGTCTTT	1015
	CAATGATCAACCAAACCCAC	
*PEDV-S5*	TAGCTTCTCTGCCCAATAGA	371
	AGCTTCGTAAGGTTGAAGTC	
*PEDV-N*	CAAACGGGTGCCATTATCTCT	901
	TTCGACAAATTCCGCATCTCC	
*TGEV-S*	GTTTTCACTCGCAAATGCAG	571
	CACACATACCAAGGCCAT	
*PoRV-VP6*	GAACATGTCGTGCCATTG	257
	GTGGTCGTACTAGCTGAAAT	
*PoSaV-VP_1_*	GTGATGTGGTTGAGAATGTG	510
	CATAGGTGAAAGTGGTGTCT	
*PaStV-0RF_2_*	AGAAGGAGAAAACAGAGCAAG	693
	TTTTGTATCTTCGCCCTTGA	
*PDCoV-N*	TCGTGTTACTTGGGTTAAGG	636
	TCTTGTTTGTCAGGCTTCTT	

**Table 2:** Detailed information about the downloaded full-length sequences of the PEDV S gene.

Strains	Accession	Countries	Year	Strains	Accession	Countries	Year
CV777	AF353511	Switzerland	1978	AJ1102	JX188454	China	2011
CV777	JN599150	China	1994	JS2008	KC109141	China	2008
CH/HN2247	KX981440	China	2016	PEDV4-S-3	KT313038	China	2015
KNV-1305	KJ662670	South Korea	2014	USA/indiana/17835	KF452323	USA	2013
K14JB01	KJ623926	South Korea	2014	HBHG1	KY775045	China	2016
LNBX-2018	MT294132	China	2018	VN/JFP1013-1	KJ960178	Vietnam	2014
CH/GZJP/2017	MH593138	China	2017	GDhz18	MN368716	China	2019
USA/Minnesota	KR265843	USA	2014	CH/SCCZ/2018	MH593148	China	2018
CH/SCNJ-1	MW145535	China	2020	USA/Missouri102	KJ645693	USA	2013
CH/SCQL-1	MN617866	China	2018	swun-Y1-SCCQ	MK820041	China	2019
OH851	KJ399978	USA	2014	SM98-5P	KJ857455	South Korea	1998
JS-HZ2012	KC210147	China	2012	KNU-1406-1	KM403155	South Korea	2014
CHGD-01	JX261936	China	2011	YnP7	MG334008	China	2017
HK2021	OL762457	China	2021	SC2021	OL411879	China	2021
SC-ZY-1	KR732651	China	2014	SX-TY1/2017	MN412571	China	2017
VN97/HN/2013	HX982561	China	2013	GDsg16-1	MN368690	China	2016
FGE-20140427	KJ777677	China	2014	CH/JLDH/2016	MF346935	China	2016
DR13	JQ023162	South Korea	2011	PC21A	KR078299	USA	2013
KNU-0801	GU180142	South Korea	2009	USA/Colorado/2013	KF272920	USA	2013
83P-5	AB548618	Japan	2010				

**Table 3:** Detailed information about the fecal samples over the past decade.

City	Year	Weaned Piglet	Nursery Pig	Fattening Pig	Total	Representative Epidemic Strains
Zhaotong	2022, 2016	35	100	508	643	
Dali	2022, 2018, 2017, 2015, 2014	21	20	85	126	YN-DL-2107, YN-DL-2207
Yuxi	2022, 2020, 2021, 2015	59	24	0	83	YN-YX-1903, YN-YX-2003, YN-YX-2203
Chuxiong	2022, 2017	45		127	172	YN-YM-2003, YN-YM-2206, YN-YM-22061, YN-WD-2206, YN-WD-22061, YN-WD-22062
Qujing	2022, 2019, 2015	174	78	40	292	YN-QJ-1412, YN-QJ-1507, YN-QJ-2210, YN-QJ-22101, YN-LL-1603
Baoshan	2022	0	0	15	15	YN-BS-1807, YN-BS-1912
Kunming	2022, 2016, 2015	57	25	146	228	YN-KM-1404, YN-KM-1503, YN-KM-1705, YN-KM-1706, YN-SM-1508
Honghe	2016, 2013	77	13	90	180	YN-HH-1602
Lincang	2022, 2018	0	0	65	65	YN-LC-1803
Lijiang	2022	22	0	0	22	YN-LJ-2112, YN-LJ-2203
Wenshan	2022, 2016	25	0	0	25	YN-WS-1603
Total	2013~2022	515	260	1076	1851	

**Table 4:** Population and seasonal distributions of PEDV in Yunnan Province.

Population/Season	Sample Size	Positive Cases	Detection Rate
Fattening Pig	1076	93	8.64%
Weaned Piglet	515	321	62.3%
Suckling Sow	260	136	52.3%
Spring	447	275	61.5%
Summer	765	97	12.7%
Fall	59	249	23.7%
Winter	390	129	33.1%

**Table 5:** Comparison of amino acid difference sites between Yunnan strains and CV777 strain.

Mutation Site	Amino Acid Mutation	Mutation Site	Amino Acid Mutation	Mutation Site	Amino Acid Mutation
58	S→L	123	S→L	127	F→S
166	A→V	218	P→L	220	I→S
237	Y→C	240	T→I	247	K→R
290	P→H	302	P→L	334	S→P
343	E→Q	389	I→T	412	Q→L
414	S→F	428	R→K	462–463	PS→LF
471	T→M	504	I→T	527	L→F
584	R→S	687	N→D	719	R→W
721	Q deletion	744	S→P	772	E→G
832	P→L	852	T→I	862	T→M
906	L→Q	911	R→H	922	A→V
932	T→I	941	R→C	945	L insertion
959	R→L	1026	S→A	1029	N→T
1146	L→I	1155	G→D	1214	S→R
1259	Y→H	1265	S→F	1283	H→Y
1284	I insertion	1285	Q deletion	1286	H→Y
1291	C→S	1303	H→Y	1313–1314	SL→LF
1339	V→A				

**Table 6:** Comparison of amino acid difference sites between Yunnan strains and AJ1102 strains.

Mutation Site	Amino Acid Mutation	Mutation Site	Amino Acid Mutation	Mutation Site	Amino Acid Mutation
453	H→L	462	P→L	579	S→F
875	S→L	902	A→V	911	R→H
947	Q→L	959	R→L	983	I→T
1259	Y→H	1280	S→P		

## Data Availability

The gene sequences of the PEDV S gene obtained in this study have been deposited in GenBank under the accession numbers YN-KM-1404 PP923928; YN-QJ-1412 PP923929; YN-SM-1508 PP923930; YN-KM-1503 PP923931; YN-QJ-1507 PP923932; YN-HH-1602 PP923933; YN-WS-1603 PP923934; YN-LN-1603 PP923935; YN-KM-1705 PP923936; YN-KM-1706 PP923937; YN-LC-1803 PP923938; YN-BS-1807 PP923939; YN-BS-1912 PP923940; YN-YX-1903 PP923941; YN-YX-2003 PP923942; YN-YM-2003 PP923943; YN-DL-2107 PP923944; YN-LJ-2112 PP923945; YN-LJ-2203 PP923946; YN-WD-2206 PP923947; YN-WD-22061 PP923948; YN-WD-22062 PP923949; YN-YM-2206 PP923950; YN-YM-22061 PP923951; YN-DL-2207 PP923952; YN-YX-2203 PP923953; YN-QJ-2210 PP923954; and YN-QJ-22101 PP923955.
